# ﻿Novel brood-site pollination mutualism between sympetalous *Heterosmilax* (Smilacaceae, Liliales) and a cecidomyiid gall midge (Cecidomyiidae, Diptera) breeding in fallen male flowers

**DOI:** 10.3897/zookeys.1234.146453

**Published:** 2025-04-22

**Authors:** Makoto Kato, Atsushi Kawakita

**Affiliations:** 1 Graduate School of Human and Environmental Studies, Kyoto University, Sakyo, Kyoto 606-8501, Japan Kyoto University Kyoto Japan; 2 The Botanical Gardens, Graduate School of Science, The University of Tokyo, Tokyo 112-0001, Japan The University of Tokyo Tokyo Japan

**Keywords:** *Dasineura*, dioecy, obligate pollination mutualism, sympetaly

## Abstract

*Heterosmilax* is a unique dioecious genus of Smilacaceae (Liliales, Monocotyledon) in that both male and female flowers are sympetalous, ellipsoid, and almost closed. Our field observations in the Ryukyu Islands of Japan showed that *H.japonica* flowers are visited and pollinated exclusively by females of one cecidomyiid gall midge species, whose larvae breed in fallen male flowers and feed initially on pollen and later on floral tissue. This is the first example of obligate gall midge-associated brood-site pollination mutualism in which the pollinator brood site is fallen male flowers. The pollinator gall midge is described as *Dasineuraheterosmilacicola***sp. nov.** (tribe Dasineurini, supertribe Lasiopteridi). A molecular phylogenetic analysis reveals that it derived from a flower parasite or flower-bud galler. The sympetalous ellipsoid male flowers are thought to have adapted to allow pollen dusting on the post-abdomen of the pollinator midge, in addition to protecting and incubating internal pollinator larvae in the fallen flowers.

## ﻿Introduction

In plant–insect pollination mutualism, pollinators visit flowers to seek floral rewards, whether portable, such as nectar and pollen, or non-portable, such as floral tissue and seeds. In the typical, widespread form of pollination mutualism, pollinators collect portable rewards. However, there are also unique, always female pollinators that visit flowers and oviposit on them, such that their larvae utilize non-portable rewards. In this interaction, called brood-site pollination mutualism (Sakai 2002a), the partners are usually highly specific to each other. Especially when seeds are the reward, coevolution between the plant and the seed-parasitic pollinator under conditions of a plant–herbivore chemical arms race causes a highly specific obligate mutualism (Riley 1892; Kato and Kawakita 2017).

The insect groups identified that take part in brood-site pollination mutualism thus far include Curculionidae (Coleoptera) in *Zamia* (Zamiaceae: Tang 1987), *Eupomatia* (Eupomatiaceae: Armstrong and Irving 1990) and various genera of Arecaceae (Henderson 1986), thrips (Thysanoptera) in *Chloranthus* (Chloranthaceae: Luo and Li 1999), Phoridae (Diptera) in *Aristolochia* (Aristolochiaceae: Sakai 2002b), and Drosophilidae (Diptera) in *Nypa* (Arecaceae: Essig 1973) and several genera of Araceae (Carson and Okada 1980; Takenaka et al. 2006; Takano et al. 2012).

The gall midges (Cecidomyiidae, Nematocera, Diptera) are the smallest, but they represent the most diverse insect clade participating in brood-site pollination mutualism. Gall midges typically lay eggs in the young tissues of plants, inducing plant tissue overgrowth that provides a food source for their larvae, which grow by feeding on the induced galls (Gagné 1989). However, some species of midges oviposit on flowers without inducing plant galls; these species contribute to the pollination of the host flower, which in turn supports larval growth. This type of brood-site pollination mutualism of gall midges occurs in *Kadsura* (Schisandraceae: Luo et al. 2017, 2018), *Illicium* (Schisandraceae: Luo et al. 2010), *Siparuna* (Monimiaceae: Feil 1992), *Aspidistra* (Asparagaceae: Vislobokov et al. 2014), *Artocarpus* (Moraceae: Sakai et al. 2000; Gardner et al. 2018), and *Phyllanthus* (Phyllanthaceae: Kawakita et al. 2022; Elsayed and Kawakita 2022) (Table 1). However, in addition to brood-site pollination mutualism, gall midges contribute to pollination, such as in *Amborella* (Amborellaceae: Thien et al. 2003), *Kadsura* (Schisandraceae: Yuan et al. 2008), *Schisandra* (Schisandraceae: Yuan et al. 2007) and *Anthurium* (Araceae: Etl et al. 2022). Why various types of gall midge pollination systems have evolved several times in basal angiosperm clades (Luo et al. 2018) is unclear.

**Table 1.** A lisgt of plants taking part in brood-site pollination mutualism with gall midges.

Plant					Pollinator cecidomyiid midge			References
Order	Family	Genus	Sex expression	Brood site	Supertrribe	Tribe	Genus	
Austrobaileyales	Schisandraceae	*Kadsura* (in part)	monoecious	resin chamber of male flower	Cecidomyiidi	?	*Resseliella*	Fan et al. 2011; Luo et al. 2017
Austrobaileyales	Schisandraceae	*Illicium* (in part)	hermaphrodite	heated brood chamber	Cecidomyiidi	?	*Clinodiplosis*	Luo et al. 2010
Laurales	Monimiaceae	*Siparuna*	monoecious	male flower	Cecidomyiidi	Asphondyliini	*Asphondylia* (=*Asynapta*)	Feil 1992
Asparagales	Asparagaceae	*Aspidistra* (in part)	hermaphrodite	anther	Cecidomyiidi		not identified	Vislobokov et al. 2014
Liliales	Smilacaceae	*Heterosmilax*	dioecious	fallen male flower	Lasiopteridi	Dasineurini	*Dasineura*	This study
Rosales	Moraceae	*Artocarpus* (in part)	monoecious	fungus-infected male inflorescen	Cecidomyiidi	?	*Clinodiplosis*	Gardner et al. 2018
Malpighiales	Phyllanthaceae	*Phyllanthus* (in part)	monoecious	galled male flower bud	Cecidomyiidi	?	*Clinodiplosis*	Kawakita et al. 2022; Elsayed and Kawakita (2022)

Molecular phylogenetic studies have revealed that most of the diversity of Cecidomyiidae followed the diversification of angiosperms, and that transitions from mycophagy to phytophagy occurred only once or twice in the evolution of the subfamily (Dorchin et al. 2019). The diversification of Cecidomyiidae is reflected in the high host specificity of its genera (Carneiro et al. 2009). Among the diverse clades of gall midges, three genera of one supertribe, Cecidomyiidi (*Resseliella*, *Asphondylia* and *Clinodiplosis*), have been shown to take part in brood-site pollination (Table 1).

Recently, we found a further example of gall-midge-associated pollination mutualism, in a monocot clade growing on the islands of the Ryukyu Archipelago, Japan. Smilacaceae is a monocot family of Liliales characterized by tuberous or stoloniferous rhizomes, reticulate leaf venation, paired petiolar tendrils, radial dioecious flowers, umbellate inflorescences, fleshy berries, and a mostly woody, climbing habit (Qi et al. 2013). This family of ~210 species is widely distributed in the tropics and subtropics, but it has diversified especially in Asia and the Americas (Qi et al. 2013). Smilacaceae has been classified into two genera, *Smilax* and *Heterosmilax*, differentiated, respectively, by their schizopetalous and sympetalous ellipsoid flowers (Koyama 1984). Recent morphological and molecular phylogenetic studies, however, have shown that *Heterosmilax* is a monophyletic group within the genus *Smilax* and should be synonymized under *Smilax* (Qi et al. 2013). Schizopetalous flowers of some *Smilax* species emit a carrion-like odor and are visited and pollinated by pollen-seeking insects such as bees, beetles, and flies (Sawyer and Anderson 1998). By contrast, the pollination system of sympetalous ellipsoid flowers of *Heterosmilax* was unknown.

Our preliminary observations suggest that the *Heterosmilax* flowers are visited exclusively by cecidomyiid midges of the genus *Dasineura* and that the midge larvae breed in male flowers. *Dasineura* is a species-rich genus of Cecidomyiidae (tribe Oligotrophini, supertribe Lasiopteridi, subfamily Cecidomyiidae) comprising 476 species (Gagné and Jaschhof 2021) and generally associated with the flowers of diverse angiosperms (Gagné 1989). Thus, the newly identified interaction between *Heterosmilax* and *Dasineura* gall midges provides novel insights into how a flower parasite became a mutualistic pollinator, and how brood-site pollination mutualism has evolved in a monocot clade. In the following, we describe the pollination system of *Heterosmilaxjaponica* and report the pollinator gall midge as a new species. The phylogenetic position of the pollinator based on a molecular phylogenetic study and the evolution of pollination mutualism are discussed.

## ﻿Materials and methods

### ﻿Studied plants and field sites

*Heterosmilaxjaponica* grows along the fringes of evergreen forests in the Ryukyu Archipelago and bears flowers from March to August. Insect visits to the flowers were observed directly at Amami-Ôshima Island (Higashi-nakama: 28.2856°N, 129.4355°E, altitude 120 m), Iriomote Island (Funaura: 24.3987°N,123.8040°E, altitude 20 m, and Komi: 24.2929°N, 123.8964°E, altitude 140 m) and Yonaguni Island (Mt Kubura: 24.4572°N, 122.9586°E, altitude 90 m) and photographed using time-lapse and video cameras. Insect behavior was also observed directly. Samples of female flowers were obtained and examined for pollen attachment on stigmas and insect herbivory on flowers. Male flowers were sampled and examined for pollen production in anthers and insect infestation of pollen and petals. Because preliminary observation suggested that male flowers fall 1 day after they bloom, fallen male flowers were collected and examined for insect infestation on floral tissue.

### ﻿Observation of floral visitors

Insect visitors to male and female flowers were observed using a time-lapse camera on 4–5 June 2018 on Yonaguni Island, on 14–16 June on Amami Island, on 15–16 April 2023 at Funaura, Iriomote Island, and on 5–6 April 2024 at Komi, Iriomote Island. Some of the visitors were collected directly into killing jars for later identification and determination of pollen attachment.

After each observation, 60–200 female flowers visited by insects were collected and examined for pollen attachment on the stigmas and for deposited eggs. In addition, 200–300 male flowers visited by the insects were collected and examined for eggs/larvae of the insects. The male flowers were placed in plastic cases filled with vermiculite and kept moist in an incubator at 25 °C for about a month.

The gall midges collected on the flowers, having emerged in the rearing cases, were either pinned using micropins (stainless steel pins A1, Watkins & Doncaster Co.) and freeze-dried in a refrigerator or preserved in 99% and 70% ethanol. Some of these specimens were later dissected according to the method outlined by Gagné (1989) and then mounted on permanent microscopic slides using Euparal (Waldeck GmbH & Co. KG). Gall-midge larvae collected from male flowers were also preserved in 99% and 70% ethanol. The pupa is unknown. The specimens and slides were examined under a microscope (VHS-7000; Keyence). The terminology used to describe adult morphology followed that of McAlpine et al. (1981). The type specimens are deposited at the National Museum of Nature and Science, Tokyo (NSMT), and other specimens at Kyoto University Museum, Japan.

### ﻿Phylogeny of the pollinator gall midge

As *Dasineura* is a species-rich, polyphyletic genus (Dorchin et al. 2019), the approximate phylogenetic position of the gall midge associated with *Heterosmilaxjaponica* was investigated by sequencing the nuclear 28S ribosomal RNA gene and the mitochondrial cytochrome c oxidase subunit I (COI) gene of one adult gall midge collected on a flower at Funaura, Iriomote Island. The obtained sequences were analyzed using the 28S rRNA and COI gene dataset of Dorchin et al. (2019), who studied the phylogenetic relationships of all Cecidomyiinae (the largest subfamily of Cecidomyiidae). Two additional adults and 10 larvae collected on the flowers at Funaura were further sequenced for the mitochondrial COI gene to confirm that the adults and larvae belonged to the same species.

Genomic DNA was extracted using the NucleoSpin Tissue DNA extraction kit (Macherey-Nagel, Germany). The primers used for the PCR were D2 and D3R (Belshaw et al. 2001) and LCO and HCO (Folmer et al. 1994) for the 28S rRNA and COI genes, respectively. The PCR conditions were those described in Dorchin et al. (2019). The PCR products were purified using the ExoSAP-IT cleanup kit (Thermo Fisher Scientific), and sequencing was outsourced to FASMAC (Kanagawa, Japan). Electropherograms and multiple sequence alignments were assessed using MEGA v. 11 software (Tamura et al. 2021). Multiple sequence alignments were conducted using ClustalW, as implemented in MEGA, with default settings; obvious misalignments were corrected by visual inspection. A maximum-likelihood (ML) analysis of the concatenated 28S and COI dataset was performed using raxmlGUI 2.0 software (Edler et al. 2021). The nucleotide substitution model that best fit each gene partition was selected under default settings, and the resulting TPM3uf+I+G4 and GTR+I+G4 models were used for the 28S and COI gene partitions, respectively. Branch support was evaluated in a bootstrap analysis with 1,000 replications. Newly obtained sequences were deposited in GenBank under accession numbers PV203684 and PV199165–PV199177 (see the Suppl. material 1 for the accession numbers of all sequences used in the ML analysis).

## ﻿Results

### ﻿Pollination mutualism

**Figure 1.**
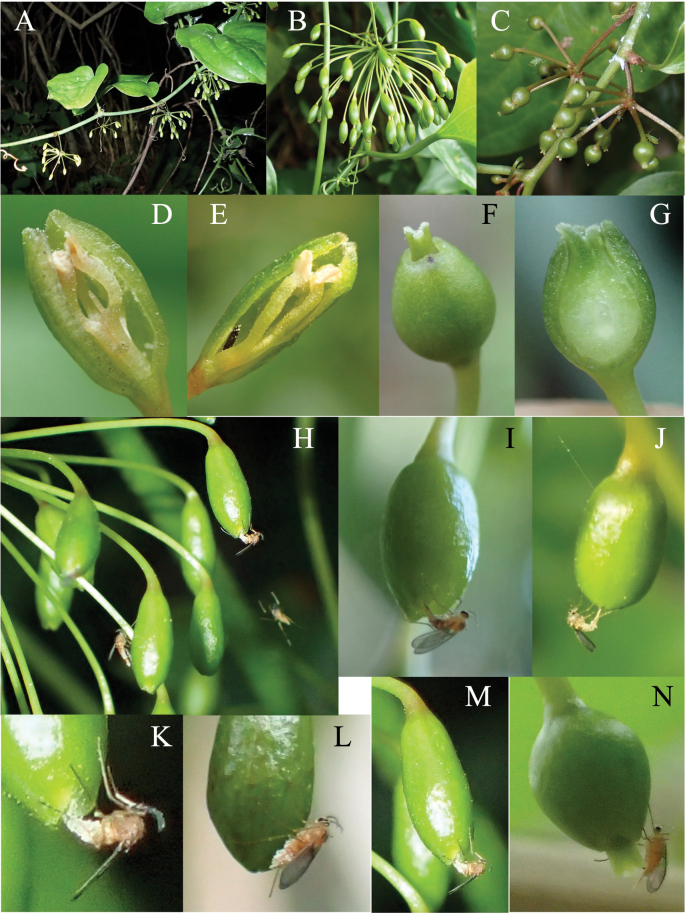
Male and female flowers of *Heterosmilaxjaponica* visited by a *Dasineura* midge **A** male plant with male inflorescences **B** male inflorescence **C** female inflorescence **D** cross section of a male flower **E** male flower visited by thrips **F** female flower **G** cross-section of a female flower **H–M** male flowers visited by *Dasineura* gall midges **N** female flower visited by a *Dasineura* midge. Observations were made on Amami-Ôshima Island (**A, C, L, N**), Iriomote Island (**D, F–K**), and Yonaguni Island (**B, E, M**).

*Heterosmilaxjaponica* is a woody, dioecious climber growing along the edges of evergreen forests, with flowers on solitary umbels at the basal leaf axils of branches (Fig. 1A). The flowering season is from March to July, but in this study male flowers were observed only in March. On each umbel, 10–30 male and 8–20 female flowers are borne on long peduncles (Fig. 1B, C). Both male and female flowers are ellipsoid, with three fused perianths: male flowers (Fig. 1B) are ellipsoid and slenderer than the ovoid female flower (Fig. 1C). A male flower has three (rarely six) stamens, whose filaments are fused at the basal 1/4–1/2 portion (Fig. 1D). At flowering, the connate perianth dehisces only at the tip, and anthers are concealed in the connate perianths. A female flower has three stigmas, which protrude slightly from the connate perianths at flowering (Fig. 1F, G). Both male and female flowers are pendent and almost closed at flowering (Fig. 1B, C).

Our observation of insect visits to *Heterosmilax* flowers showed that both male and female flowers were seldom visited by any insects, except during the early morning. Just before sunrise, male flowers started dehiscing the tip of the perianth tube. Around the same time, minute gall midges started to swarm around male *Heterosmilax* flowers, visiting them successively (Fig. 1H). The midge belongs to the genus *Dasineura* (Cecidomyiidae) and is described as a new species in the following section. Male flowers were visited only by this species of gall midge (Cecidomyiidae, genus *Dasineura*), but the gregarious visits of the midges to the flowers ended within an hour. Midge visits to *Heterosmilax* flowers were observed from 6:12 to 6:52 on 15 April 2023 at Funaura, Iriomote Island, from 7:15 to 7:33 on 6 April 2024 at Komi, Iriomote Island, and from 7:34 to 8:25 on 5 March 2019 at Mt Kubura, Yonaguni Island. Each female midge visited a newly opened pendent male flower, walked to the opening of the perianth, extended its abdomen, and then inserted it into the perianth tube from the apical opening to lay eggs. The average time spent by a *Dasineura* midge on a male flower was 109 ± 46 s on 6 April 2024 at Iriomote Island and 97 ± 41 s on 5 March 2019 at Yonaguni Island.

**Figure 2.**
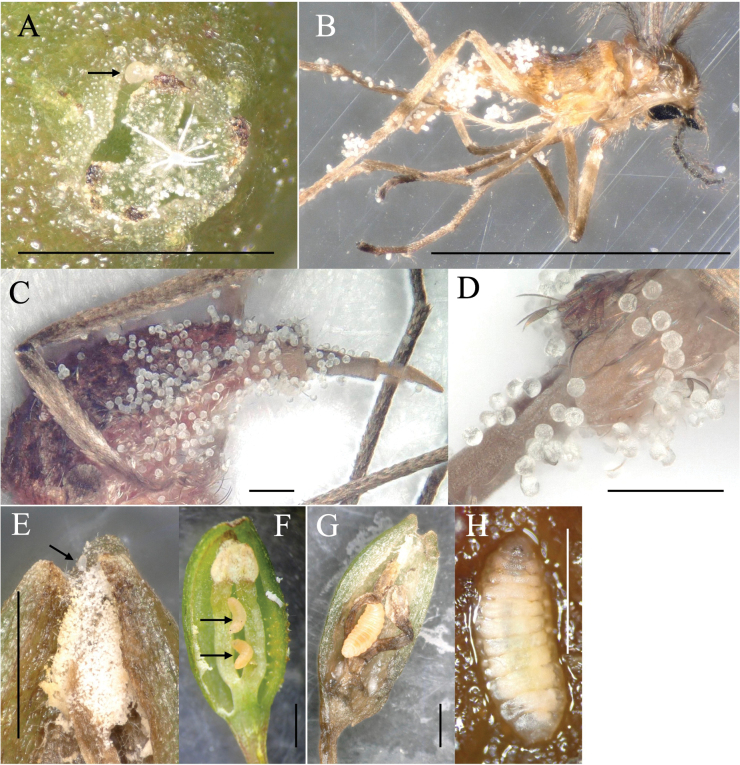
Attachment of *Heterosmilaxjaponica* pollen on the stigma and pollinator body, and larvae of the pollinator *Dasineura* midge breeding within male flowers **A** stigma of a female flower visited by the midge (arrow points to a deposited egg) **B–D***Dasineura* gall midges that have visited female flowers (**B** body **C** abdomen **D** 7–8 segments of abdomen) **E** cross-section of a male flower visited by a *Dasineura* midge (arrow points to a deposited egg) **F** two larvae (arrows) feeding on pollen in a male flower **G** third instar larva having infested pollen and floral tissue **H** midge larva in dorsal view. Scale bars: 1 mm (**A, B, E–H**); 0.1 mm (**C, D**).

All *Dasineura* midges that visited male *Heterosmilax* flowers were females, whose bodies, especially the abdomen, were dusted by plant pollen (Figs 1K, M, 2B–D). Dipteran eggs were found on the inner walls of the connate perianths of male flowers that had been visited by the *Dasineura* midge.

Almost all male flowers, together with their peduncles, fell the day after anthesis, i.e., the flowering period of a male flower is 24–36 h. Dissecting the fallen male flowers revealed that most contained one or two (rarely three) midge larvae (Fig. 2F, G), and rarely thrips (Fig. 1E). The midge larvae were initially found feeding on pollen and later infesting the perianth and filaments of the fallen flowers. Full-grown larvae were left the flowers and pupated in vermiculite in a plastic case. From the rearing case containing the flowers that bloomed on 5 March 2019, adults emerged on 29–31 March. Thus, the time spent for growth, from egg deposition to adult emergence, was 24–26 days. The emerged midges were morphologically identical to those that had visited the flower, suggesting that the midge utilizes male *Heterosmilax* flowers as a brooding site.

Female flowers were visited by the *Dasineura* midge in the evening, but its visits were rarely observed. The female midge visited female flowers, extended its abdomen, touched stigma (Fig. 1N), and engaged in pollination (Fig. 2A). While oviposition was not confirmed, eggs resembling those deposited on male flowers were observed deposited on the inner wall of the perianth tube (Fig. 2A). The infestation of female flower tissue by midge larvae was not observed.

### ﻿Taxonomy of the pollinator midge

#### ﻿ Dasineura heterosmilacicola sp. nov.

Taxon classificationAnimaliaDipteraCecidomyiidae

**Figure 3.**
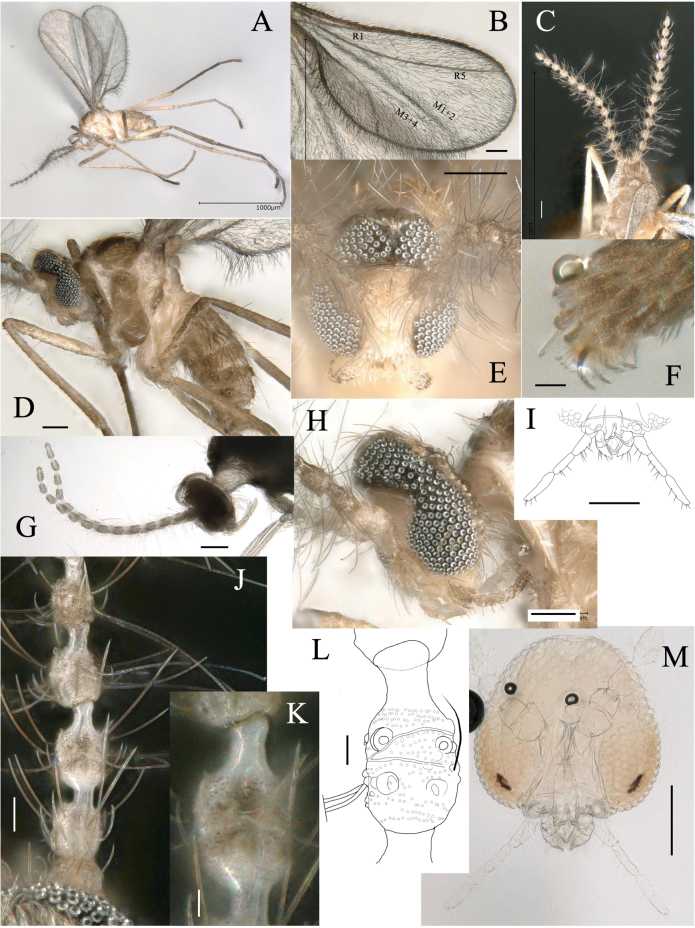
*Dasineuraheterosmilacicola* sp. nov. male **A** habitus, lateral **B** wing **C** head and thorax, dorsal **D** body, lateral **E** head, frontal **F** tarsal claw of foreleg **G** antenna, lateral **H** head, lateral **I** mouthpart, frontal **J** segments 2–6 of an antenna, dorsal **K, L** 5^th^ segment of an antenna, dorsal and lateral **M** head, frontal. Scale bars: 1 mm (**A**); 0.1 mm (**B–E, G–I, M**); 0.01 mm (**F, J–L**).

D9F1E4D9-35D0-549C-864D-97E79A292D7F

https://zoobank.org/A0D631C9-74F4-42EA-ABB3-83B4087DD40C

**Figure 4.**
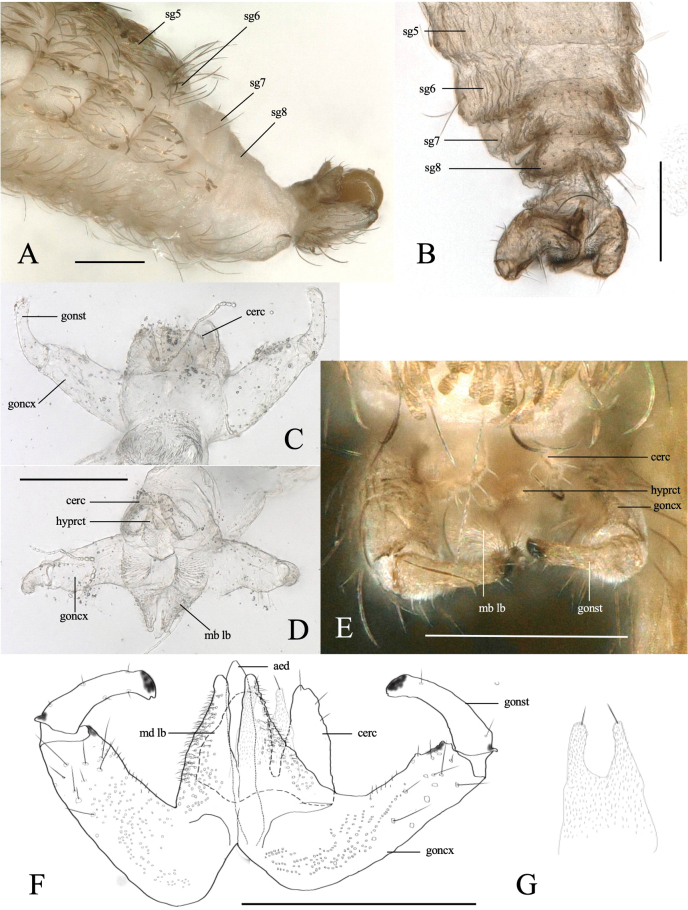
*Dasineuraheterosmilacicola* sp. nov. male abdomen **A** abdomen, lateral **B** abdomen ventral **C–F** genitalia in dorsal (**C, E**), posterior (**D**) and ventral (**F**) views **G** hypoproct. Abbreviations: aed, aedeagus; cerc, cercus; goncx, gonococxite; gonst, gonostylus; hyprct, hypoproct; mb lb, mediobasal lobe. Scale bars: 0.1 mm.

**Figure 5.**
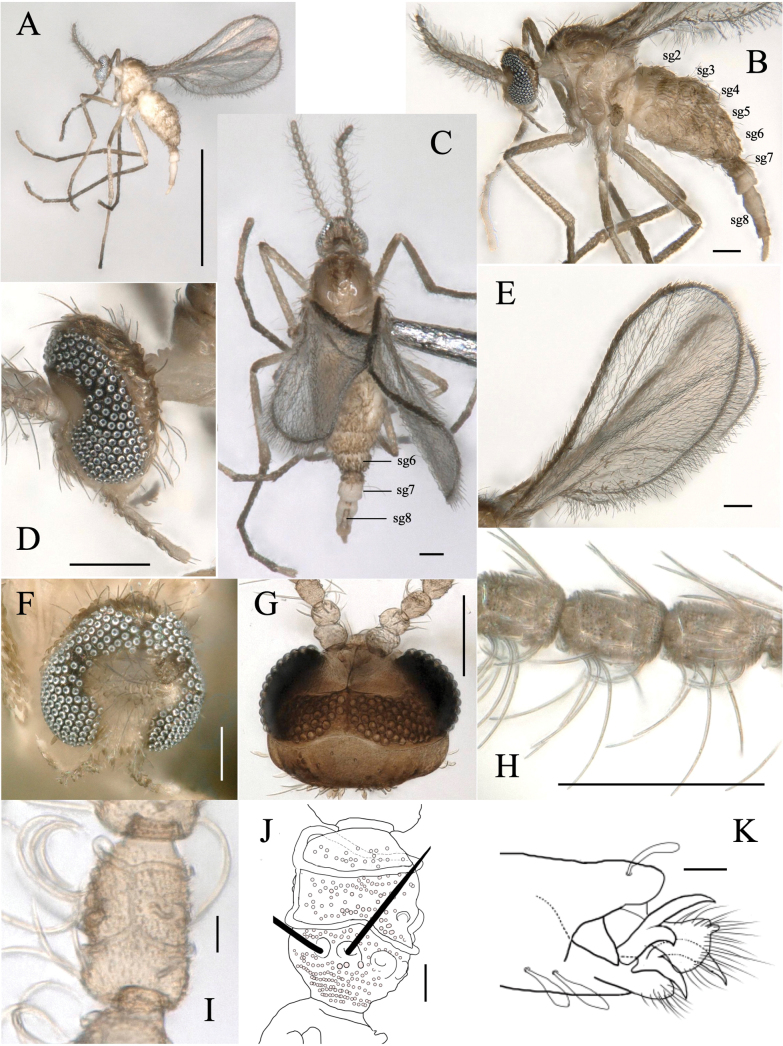
*Dasineuraheterosmilacicola* sp. nov. female **A** habitus, lateral **B** body, lateral **C** habitus dorsal **D** head, lateral **E** wing **F** head, frontal **G** head, dorsal **H** segments 3–5 of an antenna, lateral **I, J** 5^th^ segment of an antenna, dorsal and ventral **K** tarsal claw of hindleg, lateral. Abbreviations: sg2–sg8, 2^nd^–8^th^ segments. Scale bars: 1 mm (**A**); 0.1 mm (**B–H**); 0.01 mm (**I–K**).

**Figure 6.**
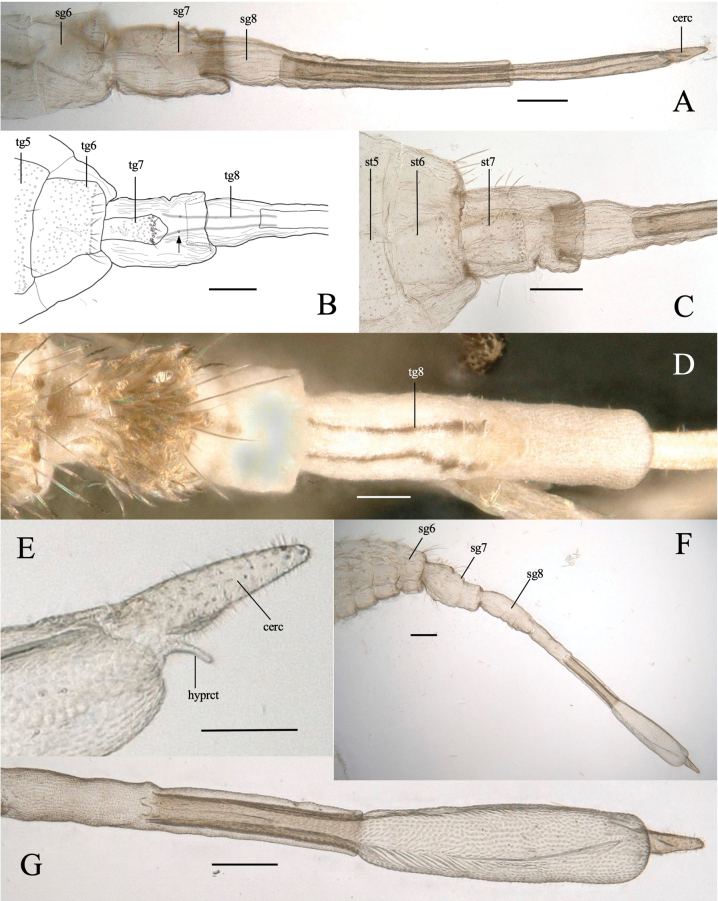
*Dasineuraheterosmilacicola* sp. nov. female abdomen **A** segments 6–8, lateral **B, C** segments5–8, dorsal and ventral **D** segments 7–8, dorsal **E** cercus, lateral **F** post-abdomen with fully protruded ovipositor, lateral **G** protruded ovipositor. Abbreviations: cerc, cercus; hyprct, hypoproct; sg6–sg8, 6–8^th^ segments; st5–st7, 5–7^th^ sternites; tg5–tg8, 5–8^th^ tergites. The arrow indicates sensilla on the 8^th^ tergite. Scale bars: 0.1 mm (**A–D, F**); 0.01 mm (**E, G**).

**Figure 7.**
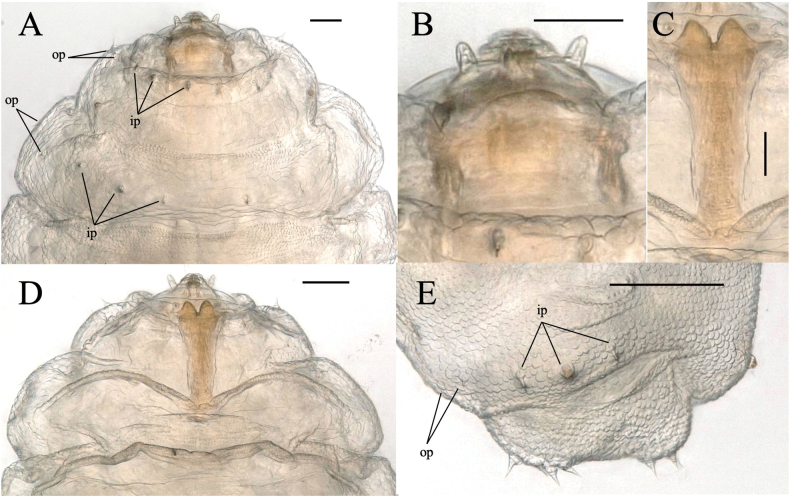
*Dasineuraheterosmilacicola* sp. nov. full-grown larva **A** head and thorax, dorsal **B** head, dorsal **C** sternal spatula, ventral **D** head and thorax, ventral **E** 8^th^ and terminal segments, left dorso-lateral. Abbreviations: ip, inner papilla; op, outer papilla. Scale bars: 0.1 mm.

Figs 3 , 4 , 5 , 6 , 7

##### Material examined.

***Holotype***: Japan • 1 ♂, NSMT-I-Dip 36246, microscopic slide; Mt Kubura, Yonaguni Island, Yonaguni-chô, Yaeyama-gun, Okinawa Prefecture; 24.4572°N, 122.9586°E; altitude 90 m; 5-III-2019 (as larva in male flower of *Heterosmilaxjaponica*), emerged on 30-III-2019; M. Kato leg.

***Paratypes***: Japan • 2 ♂ 3 ♀, NSMT-I-Dip 36241–36245, freeze-dried specimens; NSMT-I-Dip 36247, microscopic slide; same data as holotype, emerged on 30–31-III-2019 M. Kato leg. • 2 ♂ 3 ♀, NSMT-I-Dip 36248–36250, freeze-dried specimens & NSMT-I-Dip 36251–36252, microscopic slides; Funaura, Iriomote Island, Taketomi-chô, Yaeyama-gun, Okinawa Prefecture; 16-IV-2023 (as larva in male flowers), emerged on 5–8-V-2023; M. Kato leg. • 1 ♂ 2 ♀, NSMT-I-Dip 36253–36255, freeze-dried specimens); Funaura, Iriomote Island, Taketomi-chô, Yaeyama-gun, Okinawa Prefecture; 5-VI-2018 (as larva in male flowers), emerged on 19–20-VI-2018; M. Kato leg.

##### Other material.

Japan • 1 ♂ 3 ♀; same data as holotype, emerged on 30–31-III-2019 • 1 ♂ 3 ♀; Funaura, Iriomote Island, Taketomi-chô, Yaeyama-gun, Okinawa Prefecture; 5-VI-2018 (as larva on male flower), emerged on 19–22-VI-2018 • 1 ♂ 3 ♀; Funaura, Iriomote Island, Taketomi-chô, Yaeyama-gun, Okinawa Prefecture; 16-IV-2023 (as larva on male flower), emerged on 8-V-2023 • 1 ♂ 4 ♀; Higashinakama, Amami-Ôshima Island, Kagoshima Prefecture; 13-VI-2018 (as larva on male flower), emerged on 1-VII-2018; all these non-types M. Kato leg.

##### Diagnosis.

A small species (wing length 1.2–1.5 mm); antenna with 12–13 flagellomeres in males, 11–12 in females. Eyes holoptic, with a distinct constriction at the middle. Tarsal claws bifid, each strongly curved downward beyond mid length. Male gonostylus basal 1/3 swollen, apically forming a dark brown sclerotized claw. Female abdomen with segments 7–8 protrusive; extended ovipositor 9–10× as long as 7^th^ tergite; eighth tergite divided into two separate, narrow longitudinal sclerites, with a pair of anterior granular sensillae. Larva feeds on internal tissue of fallen male flower of *Heterosmilaxjaponica* (Smilacaceae).

##### Description.

**Adult male** (Figs 3, 4):

***Head*** (Fig. 3E, H, M): eyes holoptic, with a distinct constriction at the middle, along the frontal margin around antennae sockets. Eye facets circular; eye bridge 5–6 facets long. Antenna (Fig. 3C): scape and pedicel white and rounded; pedicel 2/3 as long as the scape (Fig. 3H); flagellomeres12–13, brownish, with short, naked neck; neck length about 1/4 as long as node; circumfila composed of a continuous sub-basal band joined with a partial subapical band; 13–15 long subapical and 12–14 short sub-basal setae with enlarged alveoli (Fig. 3G, J–L). Palpus 4-segmented; segments 2–3 of similar length, 1.7 times as long as the 1^st^ and 0.66 times as long as the 4^th^ (Fig. 3I, M); each segment with several strong setae and covered by brownish scales (Fig. 3H).

***Thorax***: wing (Fig. 3B) length 1.3–1.4 mm; R1 joining C before mid-length of wing; R5 curving anteriorly and joining C before wing apex. M3+4 connected with Cu, forming a fork. Wing membrane with dense, dark microtrichia. Halter brownish. Scutum, scutellum, mediotergite, propleuron, anepisternum, katepisternum, and katatergite brown; other parts whitish (Fig. 3D). Anepisternum with 5–6 setae on dorsal third; anepimeron with 7–8 setae; remaining pleura bare.

Legs slender and brown, but inner sides paler. Tarsal claws bifid on all legs; each claw strongly curved downward beyond mid-length; empodia as long as tarsal claws (Fig. 3F).

**Figure 8.**
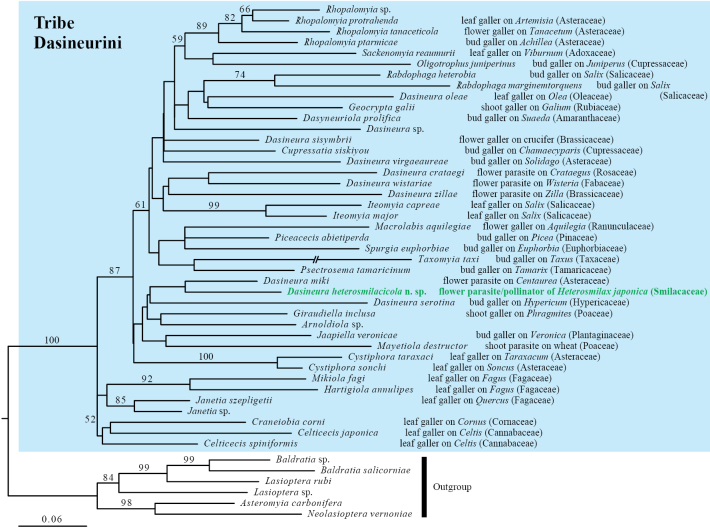
Maximum-likelihood tree of the gall midge tribe Dasineurini based on combined 28S rRNA and COI gene sequences, showing the phylogenetic position of the *Dasineura* gall midge that pollinates *Heterosmilaxjaponica*. Numbers above the branches are bootstrap values based on 1,000 replications. Sequence data are from Dorchin et al. (2019), except the newly obtained sequence for the pollinator of *Heterosmilaxjaponica*. The long terminal branch of *Taxomyiataxi* was trimmed.

***Abdomen***: tergites 1–6 rectangular, each with a single row of setae along posterior margin and lateral setae, elsewhere mostly covered with brownish scales; 7^th^ tergite unsclerotized, with a pair of medial setae (Figs 3D, 4A); 8^th^ tergite unsclerotized. Sternites 1–7 rectangular, divided transversely, sclerotized as two pigmented transversal bands, each bearing a row of setae; 8^th^ sternite smaller than others, emarginate posteromedially (Fig. 4A, B), setulose.

Terminalia (Fig. 4C–G): gonocoxite stout cylindrical, setulose, with setae on apical half and densely setose inward. Gonostylus tapering distally, weakly arched inward, sparsely setulose, apically forming dark-brown sclerotized claw. Mediobasal lobe subdivided, sheathing aedeagus, slightly shorter than aedeagus, densely covered with setulae directed backward. Hypoproct shorter than cerci, with narrow lobes, U-shaped incision about 1/3 length of hypoproct, uniformly covered with microtrichia and with one apical seta on each lobe (Fig. 4G). Cerci ovate, deeply separated, setose distally. Aedeagus with subtriangular apex.

**Adult female** (Figs 5, 6)

***Head***: similar to male except the antenna. Antenna (Fig. 5C); scape and pedicel pale and rounded, pedicel 2/3 as long as scape (Fig. 5D, G); 11–12 flagellomeres (Fig. 4C), brownish without neck; circumfila composed of a continuous sub-basal band and a partial subapical band joined by two connectives: dorsally with 6–7 basal setae with large alveoli; ventrally with 6–7 long subapical and 6–7 short sub-basal setae with enlarged alveoli (Fig. 5I, J).

***Thorax***: wing (Fig. 5E) length 1.2–1.5 mm. Wing venation similar to male. Notum pale brown with a pair of dark longitudinal stripes covered by long setae and brownish scales (Fig. 5C). Legs similar to male (Fig. 5K).

***Abdomen***: tergites 1–6 rectangular, 5^th^–6^th^ each narrower than the previous one; all with single row of posterior setae, elsewhere mostly covered with scales (Fig. 5B, D); 7^th^ tergite narrow, with many setae on posterior margin, covering only the anterior half of 7^th^ segment; posterior half naked without scale; 8^th^ tergite divided into two separate, narrow longitudinal sclerites; sclerites slightly divergent anteriorly and subparallel posteriorly, with a pair of anterior granular sensillae (Fig. 5B). Sternites 1–7 rectangular, divided transversely, sclerotized as two pigmented transversal bands, each bearing a row of setae (Fig. 6C). Long tubular ovipositor, usually housed in segments 6–8, but protruding and extended at oviposition (Fig. 6F, G); extended ovipositor (from base of 8^th^ segment to cercus apex) 9–10× as long as 7^th^ tergite. Cerci as long as 7^th^ tergite, fused medially into a single terminal lamella, evenly microtrichose; hypoproct narrow, microtrichose, with a pair of distal setae (Fig. 6E).

**Larva.** full-grown larva (Figs 2H, 7): yellowish white, cylindrical, slightly flattened dorso-ventrally, pointed anteriorly, blunt posteriorly (Fig. 2H). Head capsule hemispherical, cephalic apodemes about as long as head capsule, antennae about twice as long as wide (Fig. 7A, B). Sternal spatula anteriorly bidentate with V-shaped emargination, slightly extended laterally just posterior to teeth; length/width ratio is 3–4 (Fig. 7C, D). Thoracic and abdominal segments dorsally with three inner and two outer lateral papillae on each side; each papilla with seta, except the central inner papilla (Fig. 7A, E). Terminal segment dorsally with eight terminal papillae, each with seta (Fig. 7E).

##### Etymology.

The name *heterosmilacicola* denotes living on *Heterosmilax*.

##### Japanese name.

Karasukibasankirai-hana-tamabae.

##### Host plant.

Male flower of *Heterosmilaxjaponica* (Smilacaceae).

##### Biological notes.

An adult female visits a male flower of the host plant species and lays an egg in the perianth tube. The larva grows by feeding on the pollen and the floral tissue of the fallen male flower. This species is the obligate pollinator of the host plant, breeding in fallen male flowers.

##### Distribution.

Japan: Ryukyu Archipelago.

##### Remarks.

So far, 10 *Dasineura* species are known from Japan (host plant: Pinaceae 3 spp., Fabaceae 3 spp., Symplocaceae, 1 sp., Rubiaceae 1 sp., Asteraceae 1 sp., Adoxaceae 1 sp.: Yukawa 1971; Elsayed et al. 2017). No species has been collected from monocots, and there are no described species closely related to this one. The species resembles *D.wisteriae*, which induces gall formation on flower buds of *Wisteria* (Fabaceae) (Nakawatase and Yukawa 1984), but there are fewer flagellomeres than in the latter (male: 12–13 vs 14–15), and the life cycle is multivoltine (the latter univoltine). This species most closely resembles *D.camassiae*, whose larva grows in the flower bud galls of two monocot species of *Camassia* (Asparagaceae, Agavoideae), but is discriminated from the latter by the number of flagellomeres (male: 12–13 in this species, 13–15 in the latter; female: 11–12 in this species, 13–14 in the latter), the morphology of the male terminalia (cerci deeply incised in this species, but shallowly incised in the latter), the morphology of the female 7^th^ tergite (subparallel in the former, but distinctly constricted at the middle in the latter), and the larval morphology (tergite dorsally in each side, with five papillae in the former and four in the latter; length/width ratio of sternal spatula is 3–4 in this species and 7–8 in the latter).

### ﻿Phylogeny of the pollinator gall midge

Molecular phylogenetic analysis of the 28S rRNA and COI genes revealed that the pollinator midge is closely related to *Dasineuramiki*, a flower parasite on Asteraceae (Fig. 8). The 615-bp COI sequences of the three adults and 10 larvae were all identical, except for one adult and one larval sequence that differed by a single base, confirming that all sampled adults and larvae belonged to a single species.

## ﻿Discussion

### ﻿Brood-site pollination mutualism

A mutualistic interaction between *Heterosmilaxjaponica* and a newly identified flower-parasitic gall midge, *Dasineuraheterosmilacicola* sp. nov., which breeds in the male flowers, was observed on several islands of the Ryukyu Archipelago, Japan. This example of brood-site pollination mutualism associated with a gall midge is the second to be observed in monocots, and the first report of mutualism associated with the cecidomyiid supertribe Lasiopteridi (Table 1). Similar to other examples of gall-midge-associated brood-site pollination mutualism, *Heterosmilax* has unisexual flowers, and pollinator larvae breed only in fallen male flowers (Table 1).

In contrast to other descriptions of mutualism, the female pollinator gall midge identified in this study visited male flowers almost exclusively during the early morning, and male flowers fell the day after anthesis, whether or not they had been visited by gall midges. When the post-abdomen of a female midge is inserted into a male ellipsoid flower from the narrow flower entrance, it becomes dusted with pollen. The fallen male flower must therefore be the brood site for the pollinator gall midge larvae, with the larvae initially feeding on pollen and later on floral tissue. Thus, the sympetalous ellipsoid flower seems to have adapted to allow pollen dusting on the elongated post-abdomen of the female gall midge while also protecting and incubating gall midge larvae. This example of brood-site pollination mutualism therefore differs from other mutualisms in the sexual expression of the flowers, the morphology and persistence of the male flowers, the brood site for pollinator larvae, and the food of those larvae.

As a female gall midge that has visited female flowers is dusted with pollen (Fig. 2B–D), it acts as a potential pollination agent if other such midges also visit female flowers. While visits to female flowers were rarely observed (Fig. 1N), the eggs that were presumably laid by the female gall midges were often deposited on pollinated flowers (Fig. 2A), suggesting that female flowers also attract female gall midges via floral odor by deceit. The absence of larvae on female flowers suggests that larvae are unable to grow in female flowers. Rather, female gall midges visit both male and female *Heterosmilax* flowers to oviposit, with the deposited eggs able to grow only on fallen male flowers. In addition, the observation that *Heterosmilax* flowers on all three islands (Yonaguni, Iriomote, and Amami-Ôshima) were visited exclusively by the same gall midge species suggests that the mutualism is highly specific and obligate.

Because male flowers contribute to the production of pollinator gall midges, pollination efficiency presumably depends on their abundance. The flowering season of *Heterosmilax* is long, lasting up to five months (from March to July), and only male flowers bloom during the early flowering season. This flowering pattern can be understood as reproductive strategy of male plants.

*Heterosmilax* is monophyletic (Qi et al. 2013) and comprises 11 species found in Southeast and East Asia (Koyama 1984). Further studies on other congeneric species will reveal whether sympetalous ellipsoid flowers have evolved to allow similar brood-site pollination mutualism, and whether parallel cospeciation has occurred in the plant and pollinator lineages.

### ﻿Evolution of the pollinator gall midge

The pollinator gall midge is a newly discovered species within the diverse genus *Dasineura*; it is also the first pollinator species recognized within the tribe Dasineurini and the supertribe Lasiopteridi. All previously known pollinating gall midge species belong to the supertribe Cecidomyiidi (Table 1).

The phylogenetic tree (Fig. 8) suggests the evolution from flower-galler/parasite to mutualistic flower parasite on *Heterosmilax* flowers. For example, *D.camassiae* is a flower bud galler of *Camassialeichtlinii* (Asparagaceae, Agavoideae) that reduces the fitness of the host plant by feeding on the ovules of bisexual flowers (Gagné et al. 2014). A comparison of the two *Dasineura* species shows that tergites 6 and 7 are much wider in *D.heterosmilacicola* than in *D.camassiae*, such that the post-abdomen is covered by larger numbers of setae and scales, which may promote pollen attachment. *D.camassiae* females oviposit on the flower buds of the host plant, with the larvae that leave the bud galls entering hibernation until the next flower season, in spring, indicative of a univoltine life cycle. *Dasineuraheterosmilacicola*, by contrast, is multivoltine, with several generations occurring during the long (up to 5 months) flowering season.

The timing of flower visitation would be expected to differ between parasitic and mutualistic gall midges. Many females of *D.heterosmilacicola* were seen to gregariously visit newly opened male flowers in the morning, while *D.camassiae* visits flower buds before anthesis. Further studies on the temporal changes in floral odor from flower bud formation to anthesis and the corresponding responses of gall midges are needed. Furthermore, assuming that the genus *Dasineura* is highly diverse, reflecting its associations with a diverse group of angiosperm flowers, comparisons based on phylogenetic relationships will provide insights into the transition from flower parasites to brood-site pollinators.

## Supplementary Material

XML Treatment for Dasineura heterosmilacicola

## ﻿Additional information

### Author ORCIDs

Makoto Kato https://orcid.org/0000-0002-0602-7082

Atsushi Kawakita https://orcid.org/0000-0002-4864-7423

### Data availability

All of the data that support the findings of this study are available in the main text or Supplementary Information.

## Supplementary materials

Supplementary material 1 Taxa used in the phylogenetic analysis, with GenBank accession numbers Data type docx Explanation note Sequences of *Dasineuraheterosmilacicola* sp. nov. (shown with asterisk) were newly obtained; other sequences are from Dorchin et al. (2019). https://binary.pensoft.net/file/1313263This dataset is made available under the Open Database License (http://opendatacommons.org/licenses/odbl/1.0/). The Open Database License (ODbL) is a license agreement intended to allow users to freely share, modify, and use this Dataset while maintaining this same freedom for others, provided that the original source and author(s) are credited.Makoto Kato, Atsushi Kawakita
