# Supplementary material for: ﻿Novel brood-site pollination mutualism between sympetalous Heterosmilax (Smilacaceae, Liliales) and a cecidomyiid gall midge (Cecidomyiidae, Diptera) breeding in fallen male flowers
Source: Zookeys. 2025 Apr 22;1234:397–416. doi: 10.3897/zookeys.1234.146453 (PMC12041868; doi:10.3897/zookeys.1234.146453)
Supplement: Supplementary material 1 — Taxa used in the phylogenetic analysis, with GenBank accession numbers [file zookeys-1234-397_article-146453__-s001.docx]

| **Supplementary Table.** Taxa used in the phylogenetic analysis, with GenBank accession numbers. Sequences of *Dasineura heterosmilacicola* sp. nov. (shown with asterisk) were newly obtained; other sequences are from Dorchin et al. (2019). | | | |
| --- | --- | --- | --- |
| Tribe | | Accession no. | |
|  | Species | COI | 28S |
| **Dasineurini** | |  |  |
|  | *Arnoldiola* sp. | MN191248 | MN201204 |
|  | *Celticecis japonica* | MN191271 | MN201225 |
|  | *Celticecis spiniformis* | MN191272 | MN201226 |
|  | *Craneiobia corni* | MN191278 | MN201231 |
|  | *Cupressatia siskiyou* | MN191279 | MN201232 |
|  | *Cystiphora sonchi* | MN191280 | MN201233 |
|  | *Cystiphora taraxaci* | MN191281 | MN201234 |
|  | *Dasineura crataegi* | MN191364 | MN201235 |
|  | *Dasineura heterosmilacicola** | PV199165 | PV203684 |
|  | *Dasineura miki* | MN191369 | MN201237 |
|  | *Dasineura oleae* | MN191282 | MN201238 |
|  | *Dasineura serotina* | MN191283 | MN201239 |
|  | *Dasineura sisymbrii* | MN191284 | MN201240 |
|  | *Dasineura virgaureae* | MN191365 | MN201241 |
|  | *Dasineura wistariae* | MN191287 | MN201242 |
|  | *Dasineura zillae* | MN191288 | MN201243 |
|  | *Dasineura* sp. | MN191285 | MN201236 |
|  | *Dasyneuriola prolifica* | MN191289 | MN201244 |
|  | *Geocrypta galii* | MN191295 | MN201251 |
|  | *Giraudiella inclusa* | MN191296 | MN201252 |
|  | *Hartigiola annulipes* | MN191300 | MN201257 |
|  | *Iteomyia capreae* | MN191303 | MN201260 |
|  | *Iteomyia major* | MN191304 | MN201261 |
|  | *Jaapiella veronicae* | MN191306 | MN201263 |
|  | *Janetia szepligeti* | MN191308 | MN201265 |
|  | *Janetia* sp. | MN191307 | MN201264 |
|  | *Macrolabis aquilegiae* | MN191321 | MN201278 |
|  | *Mayetiola destructor* | EU375697 | MN201280 |
|  | *Mikiola fagi* | MN191323 | MN201282 |
|  | *Oligotrophus juniperinus* | MN191329 | MN201287 |
|  | *Piceacecis abietiperda* | MN191333 | MN201291 |
|  | *Psectrosema tamaricinum* | MN191336 | MN201295 |
|  | *Rabdophaga heterobia* | MN191337 | MN201296 |
|  | *Rabdophaga marginemtoquens* | MN191338 | MN201297 |
|  | *Rhopalomyia protrahenda* | MN191340 | MN201299 |
|  | *Rhopalomyia ptarmicae* | MN191341 | MN201300 |
|  | *Rhopalomyia* sp. | MN191342 | MN201301 |
|  | *Rhopalomyia tanaceticola* | MN191343 | MN201302 |
|  | *Sackenomyia reaumurii* | MN191345 | MN201303 |
|  | *Spurgia euphorbiae* | MN191348 | MN201308 |
|  | *Taxomyia taxi* | MN191362 | MN201320 |
|  |  |  |  |
| **Outgroups** | |  |  |
| **Alycaulini** | |  |  |
|  | *Asteromyia carbonifera* | MN191258 | MN201215 |
|  | *Neolasioptera vernoniae* | MN191327 | MN201285 |
|  |  |  |  |
| **Lasiopterini** | |  |  |
|  | *Baldratia salicorniae* | MN191262 | MN201217 |
|  | *Baldratia* sp. | MN191263 | MN201218 |
|  | *Lasioptera rubi* | MN191313 | MN201271 |
|  | *Lasioptera* sp. | MN191314 | MN201272 |
